# The Unpublished MSS. of the Late Alfred Wigan, M.D.

**Published:** 1849-10-01

**Authors:** 


### THE JOURNAL

OF

### PSYCHOLOGICAL MEDICINE

AND

### MENTAL PATHOLOGY.

OCTOBER 1, 1849.

### Analytical Reviews.

Art. I.—*The Unpublished MSS. of the late Alfred Wigan, M.D., Author of the "Duality of the Mind," &c.*

It is difficult to form anything approaching to an accurate estimate of the loss which has been sustained by the somewhat premature death of the late Dr. Wigan. How is it possible to calculate, within our own time, the importance of the great psychological truths which he enunciated? Generations may roll away ere a just appreciation will be made of the suggestions contained in his celebrated treatise on the "Duality of the Mind." England is not so rich in medical psychologists that such a man can pass from among us without giving rise to serious and painful reflections. Without desiring for a moment to exaggerate the mental calibre of Dr. Wigan, or to form an extravagant idea of the extent of his erudition, we feel that we are only echoing the sentiments of the profession, and particularly of those who had the high privilege and honour of his personal friendship, when we observe that this physician possessed a mind of no ordinary standard. No person could be long in his society without being impressed with the conviction that he was gifted with no inconsiderable powers of thought, reflection, and observation. He had not passed through the world, but had lived *in* it. He not only *saw* but *observed.* He occupied during the earlier part of his life the agreeable position of a "travelling physician," and he had thus afforded to him ample opportunities of becoming a citizen of the world, and of thoroughly acquainting himself with men and things.

His knowledge of the human mind and heart was great. His capability of accurately estimating character was generally admitted. He was undoubtedly a man of original thought, and, like his fellows in this respect, he occasionally allowed his originality to overstep the bounds of prudence, and at times he appeared as the advocate of a paradox. But who is without his faults? Is it not the peculiar province of genius to be erratic? Dr. Wigan must not be the exception to the general rule. It was not until he had well studied the great book of nature that this physician thought of submitting his views to the profession, and laying before the public the fruits of many years' cogitations. He was not anxious to "rush into print" before he had well considered the grounds of his belief. Having had, during the latter period of his life, the honour of his most intimate friendship, we have often heard him dwell upon the patient, cautious, and inductive process he pursued for many years, with the view of satisfying his mind as to the truth of certain views he entertained, before finally resolving to appear before the world as their expositor. It would be fortunate for the cause of truth if all took a lesson out of his book, and endeavoured to ascertain the universality of a fact before proceeding to the process of generalization. We must not be understood to have assented to all the speculations of Dr. Wigan. Combined with much valuable psychological truth, there is a fair sprinkling of fallacies; but it is not our intention to dwell upon these. It is to the beauties, the great truths, which this psychologist has developed, that we are anxious to direct public and professional attention.

It affords us much pleasure to have an opportunity of gratifying the taste of our readers in this matter. Through the politeness of Dr. Wigan's family, all his unpublished MSS. have been submitted to us for publication. We have carefully examined them, and intend to select from the documents before us the facts and observations that have a bearing upon the more abstruse, important, and practical points of medical psychology.

We feel the greater pleasure in following this course, on account of the deep interest which Dr. Wigan took in the establishment and success of this journal. Although he never wrote but one short article for it, he invariably spoke to the editor and others in the warmest terms of hope and encouragement; and if it had pleased Providence to have prolonged his valuable life, he had pledged himself to zealously co-operate with others in furthering the objects of the journal, in endeavouring to create and perpetuate a taste for the more abstruse, but not less interesting and valuable, speculations of medical metaphysics. The MSS. before us contain only desultory thoughts on various points of psychology. The subject of the education of the young occupied much of Dr. Wigan's attention. We had frequent conversations with him respecting what he termed the "motiveless crimes of the young." His views on this important subject were peculiar. During the period we had the pleasure of knowing this physician, we had several consultations with him in cases of children who manifested at a very early period the most extraordinary abnormal condition of mind, not amounting to what might be considered as insanity, but very closely allied to it. The cases referred to made a very strong impression on Dr. Wigan's mind.

Our criminal records, Dr. Wigan maintained, are full of examples of atrocious acts of wickedness, committed without any assignable motive by young persons of both sexes, between the ages of sixteen and twenty-one or twenty-two. Setting fire to houses, poisoning, wanton cruelty to animals and to children, and sometimes murder.

The ages of the culprits are generally observed to be from sixteen to eighteen with girls, from seventeen to twenty-one with boys. It is extremely rare that motiveless crimes are committed by those who are either older or younger than the ages above stated. In cases of insanity there is generally a motive, however erroneous and absurd; but the instances of wickedness, to which Dr. Wigan referred, have not the character of insanity, not even of monomania.

Previously and subsequently, as well as during this period, it is obvious that acts of equal wickedness may arise from adequate causes—jealousy, revenge, hatred, slighted affection, cupidity, and sensual and especially sexual gratification. But crimes arising from such causes (known or not known) have no connexion with the present topic, they belong to another category. It is the absolutely motiveless crime of which Dr. Wigan proposed to attempt an explanation. The instances are numerous where such persons have administered poison or set fire to the house of their employers without entertaining the slightest animosity towards them; and such instances are most numerous among domestic servants, and especially farm servants, and persons of limited intellectual development.

Two or three examples which occurred at a very early period of Dr. Wigan's career, made a strong impression on his mind. Sir Charles Bell was then occupied with his investigations which led to his great discovery of the composition of nerves. Dr. Wigan was young and sanguine, and, like most enthusiasts beginning the study of anatomy, believed that we should *catch nature in the fact*, and discover the connexion between mind and matter.

The first thing which attracted his notice in these cases of motiveless crime was, that the culprit had been subject to hæmorrhage from the nose, which, in some instances, even in males, assumed almost the periodicity of menstruation. The crime had generally been committed after a temporary cessation of the habitual discharge. There was always a dull, heavy, languid look, and in no case an animated countenance, nor one with the harsh lineaments of vice. On the contrary, the expression was often mild, placid, and good, though torpid, and in some instances the face had so amiable a character, that jurymen were tempted to resist the clearest evidence, and bring in a verdict of *Not Guilty,* on the ground that the act must have been the immediate inspiration of the devil, in a mind naturally innocent and good.

When the culprit was asked by friends or by a medical man to explain his motives—if it were said to him, "Why did you do this act of wickedness? what advantage, what gratification, what benefit to yourself or to others did you contemplate?"—the answer was, generally, "I don't know; I had no reason; I thought I would do it." Further explanation they could not give than that they felt an impulse to *do something.* What that something should be was generally decided by mere accident on the casual view of the means of doing it.

He had often endeavoured to extract a different reply, but even where the interest and sympathy he expressed for a person under the influence of an ungovernable impulse, led to a strong expression of gratitude, even where an intense remorse induced the culprit to seek for any possible means of alleviation, the answer was still the same—"I had no motive; I thought I would do it."

Now it seemed to him that this ungovernable impulse depended on a local and peculiar congestion of the brain of which he attempted an explanation; whether true or not, it was an hypothesis which harmonized perfectly with the fact, and which appeared to be established by the success of a remedy founded upon it.

It will be observed that the status Dr. Wigan spoke of did not take place at the period of puberty, but generally about two years afterwards. This is an important thing to bear in mind; but the same causes, did they exist, *might* produce it at a much earlier period.

Any man whose attention has been called to the subject, and especially a man conversant with anatomy, sitting behind a stranger whose head is not concealed by a profusion of hair, can pronounce boldly the age of the party before he sees his countenance. The change which takes place in the shape of the cranium, as well as in the bones of the face, about the age of seventeen is remarkable. Generally about sixteen this alteration commences; the countenance and the whole contour of the head lose their feminine aspect, and assume a harsher shape. The change is more or less retarded in different individuals, but the deviations are few.

At this period of life, the mind alters entirely, and almost suddenly: a change to be attributed, our author believed, to the rapid growth of the organ of thought; and if subjected at this period to proper moral and intellectual discipline, guided by a rational phrenology, all the best qualities of which that specific individual brain is capable are brought to maturity.

The consequences of neglecting this duty are recorded in the registers of our criminal courts.

An analogous state of brain, which, from the different position in life of the parties led to acts apparently dissimilar, often came to his knowledge in private life among families of the highest respectability, and where every pains had been taken to inculcate good principles by education and by example. There was, he thought, no man who has lived much in the world who could not call to mind many instances of the kind.

The spirit which Dr. Wigan spoke of is sometimes manifested in cruelty to the younger members of the family—in bold defiance of the decorum of civilized life—in wanton and unnecessary exposure to shame—in reckless disregard to the feelings of others—and in a stolid exposure to risks and evils which the slightest care might avoid, and which brought neither profit nor pleasure.

Another modification of the same feeling, where the natural disposition is good, and the mind well cultivated, displays itself in acts of foolhardy daring — taking the boldest leap, walking the nearest to a precipice, incurring the greatest risk of illness from unnecessary exposure, and an hundred other manifestations of violent animal impulse, which neither arose from a spirit of emulation nor of morbid vanity, for in the greater number of cases the things were done without a witness, and only discovered by the accidents to which they gave rise, or were acknowledged to the medical attendant in the *mollia tempora fandi*.

Nay, among the intimate friends of the author's youth, during the war in the early part of the present century, he had known acts of daring, unreasoning, audacious bravery, which excited the highest admiration and applause, yet which were not done from generous rivalry, from a love of glory, or a wish to obtain applause and admiration, nor even from the feeling of animal pugnacity, but from the same stupified, headlong instinct *to do something.* Under this temporary, constitutional impulse, they have shown a courageous defiance of danger, of which they were not capable at sixteen, and which they looked back upon at four-and-twenty with a sort of vague alarm and horror. The state of brain which led to these acts being in the former case not yet arrived, and in the latter passed over.

It frequently happens that the relief experienced from bleeding at the nose, and the intense distress produced by the compression which renders such an evacuation necessary, will induce a youth to give himself a violent blow on the nose, or ask another boy to strike him for the purpose of producing it; innumerable examples of this have been cited to us by gentlemen with whom we have conversed on the subject, and every man brought up at a public school must recollect instances of the same kind. When bleeding can be thus freely induced, the disposition seems to change instantly. This severe self-infliction has been for the sole purpose of getting rid of that distressing *impulse to do something,* which is the only definition they can give of a state of mind that renders study impossible.

To state the slow process of reasoning and observation through which Dr. Wigan arrived at his convictions, and to give the examples which afforded his premises, would occupy a volume.

His firm belief was, that the immediate cause of the state of brain previously referred to, is THE INSUFFICIENTLY RAPID ENLARGEMENT OF THE BONY CAVITY TO GIVE FREE PLAY TO THE RAPID GROWTH OF THE BRAIN. That there is a permanent state of compression more or less severe, THAT THE CONGESTION IS VENOUS, AND CHIEFLY AT THE BASIS OF THE BRAIN AND IN THE CAVERNOUS SINUSES, through which passes all the venous blood of that part.

This compression may be of every degree of intensity, from that which merely produces languor and dulness, to that which brings on fever or epilepsy; it may be indefinitely modified by medical and moral means, and is almost always controllable by art. One of the grounds of his belief that this was the true rationale of the disease, was the uniform success of a practice founded on this theory.

Youths of this age—more especially females—do not bear bleeding to any considerable extent. Hysterical symptoms are easily set up by large depletion, even in males, which mystify the diagnosis, and it requires a *large* depletion to produce *any* effect on the venous circulation of this part of the brain.

If we had access to the internal jugular vein without danger of injuring the pneumo-gastric nerve, it is probable that the loss of a very small portion of blood might suffice, without any shock to a constitution which is at that age so mutable and so *impressionable.*

Bleeding from the external jugular is the next best resource, as from its large anastomosing branch through the parotid, we do at the same time abstract from the internal jugular; this, however, can only be done by abstracting at the same time from other sources. Nevertheless, it is an excellent remedy.

In necks tolerably covered with fat, however, many men have a difficulty in bleeding from this vein, and the patient and the friends have generally a great horror of an operation which looks so like *cutting the throat.*

*Bleeding from the temporal artery does not answer the purpose,* except where the congestion is general and the habit full and vigorous; whereas, in the cases to which I specifically allude, there is often a deficiency of physical power, the congestion being rather relative than positive.

Fortunately there remains a safe, easy, and effectual remedy—leeches to the inside of the nose,—a mode believed to be uniformly successful. Two or three leeches to each nostril (the part having been previously well fomented by drawing up warm water and forcibly throwing it out again) enable us to obtain any quantity of blood we may desire to take away, and to produce an influential impression on the part, with the least possible expenditure of the vital fluid, or shock to the constitution. By leaning the head forwards the bleeding continues, and by lying down it ceases. Should this not be the case, a dossil of lint at once puts an end to it.*

The moral treatment is a separate consideration; but in order to anticipate the censure of those who might draw the inference that he considered such persons not responsible for their actions, let me add that, so far from entertaining such an opinion, he proposed as the most appropriate and effective punishment, *flogging,* or, at least, some modification of corporeal suffering; but he set his face most strongly against moral mortification, and still more strongly against solitary confinement and compulsory silence.

It requires but a moment's reflexion to be convinced that, at an age when sexual desire is most intense, solitary confinement without incessant occupation is about the most mischievous kind of punishment that could possibly be devised. It often terminates in idiocy.

* Can the congestion we speak of be influenced by the tying up of the neck in boys about this time, and the tightening of the stays in girls, either from vanity or from the increase of the bust? These things may at least aggravate the mischief, by adding general to local congestion.

The change which seems to take place in the whole mind of the individual after the free application of leeches to the nose, is equal to *any* in the animal economy. Calmness, tranquillity, and composure are accompanied by a clear view of past events, and strong remorse for the misconduct to which the state of headlong impulse had led its victim.

The above is the substance of the views propounded by Dr. Wigan. Could a more deeply interesting question occupy the patient consideration of the medical philosopher? Is there a man who has passed through the busy scenes of life without meeting with cases somewhat similar to those referred to by Dr. Wigan? The tendency to crime, manifested early in life, with or without a motive, is a subject which forces attention upon the legislature. It must seriously be considered by those whose peculiar duty it is to grapple with such questions. The following observation on the supposed existence of a spasm of the nervous fibre is of a suggestive character:—

"I am fully aware how entirely hypothetical is the idea, but myself I most firmly believe that in many of the cases of concussion of the brain, especially from blows, the fibres of the convolutions are thrown into this state of spasm, which, if its violence do not produce physical mischief, (as the spasm of the gastrocnemii, a rupture of the tendo-Achillis,) may cease as suddenly as it was produced. It may be thought a very extravagant supposition, but I also firmly believe that the sudden restoration of reason before death, alluded to by Dr. Holland, and which is not a very rare occurrence, is to be attributed to the sudden cessation of the spasm which had interfered with the exercise of the understanding. This might be only in certain fasciculi of fibres, (called organs by the phrenologists,) or in one brain only That moral causes may produce a similar state of spasm is quite conceivable—that excessive exertion of the mental faculties may also produce it is also conceivable."

The doctrine propounded by some distinguished theologists as to insanity being often the result of the influence of sin on the human mind, appears to have engaged the attention of Dr. Wigan. He observes—

"Heinroth, whose numerous writings display an extraordinary mixture of mysticism, amounting almost to positive insanity, with the soundest common sense and acute observation of facts, whose ample experience in a vast establishment for the insane must have furnished him with abundant materials for correct judgment of the different forms, causes, progress, and treatment, of mental aberration, is yet so bewildered by religious enthusiasm, as boldly to assert that *sin is the cause of mental disorder*. Confused by the mixture of insanity and reason in some of these unhappy beings, instead of seeing different and contradictory states of two minds, two organs of thought, he thinks the opposition to be between the natural mind of man and the spirit of evil. It is a horrible doctrine, yet like some other theological monstrosities, it cannot always annihilate the natural goodness of a man's disposition. It did not induce him to act according to the principles which would necessarily result from it—just as clergymen who believe in predestination will yet make great efforts to save the soul of a sinner, or the fatalist strive to escape from a conflagration. Fortunately this belief is wearing out even amongst the lowest vulgar, but that it influenced the treatment of the insane a few years ago is certain, and was one of the most powerful of the motives which subjected them to the horrible tortures that now excite general indignation. It is a subject on which I cannot write or even think with calmness, when reflecting on the atrocities which I have myself witnessed at the beginning of the present century, and which then excited no indignation—scarcely even the casual notice of a philanthropist."

The following observations on the subject of senile dementia will be read with interest:—

"The form of defective brain, commonly, but inappropriately called senile dementia, is by no means peculiar to old age, for we often see it in men of forty, who have been subjected to great anxiety, or who have indulged in sensual excesses. Nothing remains in the mind of such men but what has been studied—that is, has occupied the conjoint, continuous and uninterrupted attention of both brains, a thing now almost impracticable. The ordinary occurrences of life are forgotten immediately: a man tells a story which rests perfectly in his memory, but he forgets that he told it to the same persons not half an hour before. I remember a physician, now dead, with whom I was very intimate, who said to me, 'They tell me my memory is failing. How absurd! Why, I could at this moment repeat eight hundred lines from Homer.' And he began to inflict them upon me, forgetting that within a few hours he had twice before told me the same thing, and begun the same proof of his unfailing powers.

"It is, however, sheer waste of time to speak of a subject like this, unless there be some distinct and useful object to be obtained by it; and I now recommend, as the best means of re-establishing the power of concentration, to learn by heart pieces of oratory or of poetry, especially the former, which is a severer exercise, because the memory is not aided by rhyme. Do not be discouraged by the headach, which for a time accompanies the process; this will cease, and the sufferer will be surprised at the increase of power he will gradually acquire—a power which he will discover to be accompanied by increased mental vigour in matters quite unconnected with his studies.

"In the extreme cases, accompanied by the torpor of old age, the brain seems to be in a state resembling that produced by concussion. The sympathetic system is carrying on the business of life vicariously for the brain; but in both these examples, if a loud sound be made to draw the attention, and a question then asked in a powerful tone of voice, the brain is capable of being roused into distinct perceptions. Much observation convinces me that many aged persons are left to go into the sleep of death for want of this stimulus. There are occasions where the prolongation of the life of an old person for a single week may make the difference of poverty or competence to the survivors. I remember one case where a gentleman died at eleven o'clock on the 28th of September, and left his family in great distress, when had he lived a couple of hours more he would have been entitled to another year's income, which would have placed them in comparative ease. It is so very natural to consider it cruelty to rouse them from their state of calmness and repose, that I have been more than once out-voted on such occasions. But it is like the torpor of persons benumbed with cold; if they sleep, it is the sleep of death. One brain always 'goes out' before the other; but previous to its extinction in this gradual manner, it may obey the commands of its more energetic brother when thoroughly roused, long enough to dictate a will which may save a family from destruction. I have the satisfaction of thinking that on an occasion of this kind I was the means of conferring a very important benefit on a meritorious widow and helpless children, and defeating the hopes of a brutal and unfeeling heir-at-law."

Schoolmasters and psychologists are somewhat at variance on the question of corporal punishment. It is not our intention to enter the arena, and defend the system pursued at many establishments for the education of the young. It is a difficult point to decide whether the cane and strap can be dispensed with. On a somewhat kindred subject, Dr. Wigan propounds his views. We need not say that they meet with our cordial acquiescence.

"To subject to equal punishment the little untaught child of the streets, all whose worst animal propensities have been not merely left unrestrained, but have been cultivated into precocious perfection, while his moral sense has never had presented to it any better motive than the fear of punishment—to subject such an animal to equal punishment with another, whose conscience has been carefully cultivated, seems a violation of justice. Society ought not to permit the possibility of such neglect; and in spite of the maudlin humanity of sickly sentimentalists, these neglected beings should be shipped off to colonies, where rigid discipline, new motives, habits of industry, and careful moral cultivation, may enable the creature to grow up into a useful and moral member of society: but it is not till you have carefully presented good motives that you have strictly a right to punish severely bad actions. Having done this for some time on a consistent plan, you may justly and usefully inflict any degree of punishment which will supply the deficiency of better motives, and make the creature feel that it is his interest to conduct himself justly.

"In the case of monomaniacs, as it is the fashion to name criminals, like Oxford, both brains are not suggesting evil deeds at the same time; there is one clearly capable of controlling the other if adequate motives be presented, and fear of punishment is the strongest. They no doubt feel the morbid desire to do something wrong, but they feel also that they have the power to abstain from it; and society has the right to inflict punishment of any degree of severity short of death to serve as an example to others, and a motive to retain their self-command. One instance of severe corporal punishment will operate as an electric shock to the faculties of thousands, and rouse them from the moral torpor which lets the diseased propensity take the lead. If you punish for such things, punish severely."

The following fragment on the subject of anxiety we give without any abridgment. The author and the editor of this journal had often referred in conversation to this interesting matter.

"ANXIETY!—Is there a human breast in which this awful word fails to produce an echo?—from the youth who fears to be superseded in the affections of the object of his love, or the parent who watches with alarm the blush on the cheek of his child, lest its vividness indicate latent consumption, to the old man worn down with years and sorrow, who tries to estimate the commercial convulsions that threaten to swallow up the hard earnings of a long life of privation, and reduce him to beggary.

"To specify the subjects of this corroding care would be to enumerate all the classes of society. The man of poetical imagination might give a series of individual pictures whose vividness would excite universal despair. Like the 'single captive' of Sterne, he might so harrow up the feelings of the reader by the representation of social misery individualized, that the whole world should seem a charnel house of wretchedness, unworthy of the benevolence of the Great Being who called it into existence.

"It is hard to believe it in times of despondency and alarm; but the man who stands aloof from the turmoil of the world, and occupies the higher station of independence, knows 'that all worketh together for good;' that God does not leave to a future state the expiation of many of our errors and sins, but that even in this world they work their own punishment. If we suffer for the faults and crimes of others when acquitted by our own conscience, we must endeavour to consider the misfortunes inflicted on us as part of the moral discipline by which it is His purpose to work out our improvement and fit us for final happiness.

"This view of the case, however, is appropriately left to the clergyman. It is in the capacity of physician and man of the world that I put myself forward on the present occasion, in the conviction that it is in my power to offer important consolation to the afflicted, to show how misfortune may be best borne—how its physical and moral consequences may have their force turned aside, and be rendered comparatively innocuous—how inevitable bodily ailments may be modified or cured—how some admit of great alleviation, and some of entire removal, that even by acting on the body we may render important service to the mind, and enable it to rise elastic from the pressure that, if left alone, would have crushed it to the earth.

"It is not that I would evade the consideration of other forms of unhappiness—on the contrary, I hope, sincerely and confidently, to render a service to my fellow-creatures by showing that in all cases we may anticipate and prevent, or give considerable relief to the ailments, disorders, and diseases produced by mental causes, even when it is obviously impossible to alleviate or remove their source and origin. The mind—that is, the aggregate of the functions of the brain, (for we are not here speaking of the *soul*,) can only produce disease by some sort of action on the physical structure and functions of the body. We see, however, that as accidental injury to the body (an extensive burn or scald for example) can produce a very serious effect on the mind, so also the diseased or disordered states of body, directly caused by mental emotion, act reflexly on the functions of the brain, and very often paralyse all the efforts of the sufferer, and render him incapable of using in its full power the intellect which would have otherwise shown him a mode of extrication from his embarrassment.

"Men who have mighty cares on their mind—statesmen whose confidence of retaining their position, and further ambitious hopes of personal advancement, depend on the slender and fragile thread of popular favour, or the less capricious opinion of a monarch, or whose patriotism looks forward with honourable fear to the result of a deep-laid scheme for the advancement of their country's welfare, liable at every moment to be defeated by malevolent rivals, and the unexecuted purposes rendered suspicious to those who judge by results alone—merchants who have staked vast sums on the issue of an uncertain speculation—gentlemen of fortune who have perilled their whole possessions and their honour on the result of a horse race,—such men will, perhaps, look down with contempt on the petty details of the cares of humble life which are to be found in the following pages, but—

'—— little things are great to little men.'

"The medical philosopher looks with as much interest on the anxiety of the petty tradesman, or the publican whose wealthier neighbour is gradually depriving them of the income created by untiring industry with scanty means, as on a great leviathan of the Stock Exchange, whose vast speculations involve the fate of nations. There is as much real dignity in the sufferings of the one as the other, if sanctified by a feeling of religion. Except in so far as the wish for wealth is modified by the desire to possess the means of benevolent power and the exercise of an enlightened beneficence, the hopes, fears, motives, sentiments, and feelings of the different classes, as well as their mental and corporeal sufferings, are essentially the same; and, if regarded from the heights of pure reason and philosophy, are equally deserving of honour or contempt.

"I shall, however, generally draw my illustrations from that middle class, so numerous in this country, who, possessing property, education, and refinement, are yet engaged in the incessant labour of earning the means of *maintaining their position*—which is all that the vast majority desire; the cares of the very ambitious are objects of less interest.

"It requires no argument to prove that anxiety affects the health —it is an object of daily experience; our libraries are full of books of counsel on the subject; medical works, in the enumeration of causes of lingering disease, are crammed with cases arising from this source alone, and there is scarcely a disorder wherein this state of brain is not assigned as one of the most prominent agents in disturbing the bodily health, and establishing disease. Fevers, jaundice, gout, consumption, insanity, dyspepsia, and a hundred other diseases, are so often thus created, that it would almost appear to be the sole agent in their production. And yet with all this profusion of advice and description, I cannot call to mind a single writer who has attempted to explain the *mode* in which these innumerable effects are produced; yet, till this be clearly understood, we are not in possession of half the available means of modifying or removing them.

"The distress brought on by this inability to guide the thoughts —a frequent consequence of great anxiety—this inability to use the two brains concurrently, that is, to exercise *attention* or study, is one of the most pitiable states of mind that can be conceived. Happy those who have never had personal experience of the infliction. The utility of works of imagination is thoroughly appreciated in such cases, and the sufferer would be always reading. In following the ideas of another man he can generally leave his own intellectual organs in quiet; the discordant action of the two brains may thus subside perhaps into repose, and on resuming their duties they may have re-established the unison and consentaneity which is necessary to the tranquil exercise of the mind. On such occasions, if there be no object of tender fondness, whose soothing blandishments can turn the current of the thoughts—if a man look only with terror to the time when

> "Shall dawn the dreary morrow; and the toils,
> The cares, the ills of life, with scarcely hope
> To brighten the involving gloom, and save
> The fainting spirit,"—

"on these occasions, we feel acutely the value of such a writer as Walter Scott—a man whose *medical services,* if I may so term them, would have been cheaply purchased by the nation at the price of the largest fortune ever possessed by an individual. How many a harassed brain has been soothed by his delightful fictions—how many a lingering disease has been rendered endurable—from how many has he not diverted the dismal prospect of inevitable death—to how many an aching heart has he brought consolation and comfort, and the temporary oblivion of sorrow—how many a suicide has he prevented—and how many a bewildered brain placed in repose? Such men have their mission,—they are sent into the world by a benevolent Deity for a specific purpose, and they may be compared to the blessed medicaments which have been created for the relief of suffering. I do not hesitate to say that I attribute the recovery of many a patient to the mental composure produced by reading his admirable romances, in which there is nothing to detract from the entire satisfaction and assent of a virtuous mind."

Dr. Wigan entertained strong views on the subject of capital punishment, as well as upon the indiscriminate infliction of legal penalties. Much might be said on both these important points, but as it is our intention shortly to consider the *Science of Crime* psychologically, we defer our observations on these matters until the proper period arrives for their consideration. There is much truth in Dr. Wigan's remarks relative to the mental training of most criminals. Their hereditary predisposition to crime, their early education in crime, and want of religious and moral instruction, are points which a wise and humane legislature ought duly to consider in its award of punishments.

"The inferior animals have two brains, like man, and the intellectual portion of these brains, however defective as compared with ours, can control their natural propensities. The dog can wait for a time of safe revenge, or for an opportunity of stealing with impunity, but we have not the slightest reason to believe that they can *think of their own thoughts*, the point of mental development at which begins a responsibility for the actions. If, from naturally defective formation of the organs of thought in a human being, this degree of ratiocination is beyond his powers, surely no one would hold him otherwise responsible for his misdeeds, than we hold the spider responsible for the lingering death he inflicts upon the fly; we may still, if it be our object to preserve the flies, destroy the spider, and we may remove the barely human being where he can do no further mischief. A few years only have elapsed since it was believed that we had the right to put them to death, but a more humane practice now prevails. (In the 'Annual Register,' of the years 1784 and 1785, I observe that the number hanged in one morning at the Old Bailey varied from ten or twelve to five-and-twenty; there are numerous instances of batches of sixteen, eighteen, and two-andtwenty, strangled together, many of them for crimes which, in the present day, would be thought sufficiently punished by a few years at the hulks.) In like manner, if the individual, from the want of moral training, has never been taught the art of self-control—if he has been brought up in a moral atmosphere so polluted as to stifle the growth of moral feeling—or from long indulgence of depraved habits and propensities, has become incapable of exercising a faculty once acquired, he must be judged very differently from the man who, with all the motives to good conduct carefully cultivated, deliberately prefers vice to virtue—his immediate gratification to his future advantage—and the indulgence of his own vile passions to the welfare of society."

It appeared to Dr. Wigan that, in some of the ordinary forms of insanity—of continuous or permanent insanity, the mental condition of the patients much resembled the condition of children—sensation and perception active, but reasoning powers (judgment) defective—and often (like the absence of one sense giving greater activity to another,) that the faculties particular to childhood were more acute than when they had the full possession of their reason. Some mad people, like children, have an extraordinary power of penetrating into character, and possess a sort of freemasonry which enables them to test even the sincerity of a tone of voice with a miraculous instinct. A young child can distinguish the assiduities dictated by interested motives from those which arise from a real love of children and tolerance of their vagaries. The bland and soothing tones of the doctor impose on the mother, but the child is not deceived by them. The existence, the *status* of the child is more complete in its kind than the *status* of the adult—the faculties it possesses are more perfect than the same faculties when the reason is fully developed.

Like most young practitioners, Dr. Wigan was, in the outset of his career, exceedingly anxious to please, and we dare say that his anxiety led him, as it so often leads others, into a little exaggeration of manner. He had visited a young child not very seriously ill, and had made his inquiries in so gentle and soothing, indeed so *affectionate* a manner, that it was quite evident he had made his way into the very penetralia of the mother's heart, and that there was little chance of so kind and *good* a man (so *very* fond of children) being superseded by any other person. Whatever might be the progress of the case, he came away quite satisfied with his own skill, but with a slight inkling of self-contempt at his obsequious demonstrations, and not a very exalted opinion of the lady's understanding who had been so infatuated with them. He had scarcely descended the steps, when he was called back to hear a few more last words, and was shown into a room adjoining the children's playroom; while waiting the coming of the mother he had the satisfaction of hearing the whole scene acted over again by them; one was the mamma, another the baby, and another himself; and all his words and tones were mimicked in perfection. It was quite clear that the children who had been present at the consultation, had all seen through the sweet artifices which the mother had accepted as perfectly genuine sympathy.

So it seems to be with some of the insane; they must be treated with perfect candour and veracity. They must be answered boldly and clearly, without the least subterfuge or deception. The physician ought to be exceedingly careful never to break a promise, or hold out a delusive expectation; he must behave to them with a respect which seems to arise from a real deference for humanity, even in that humiliating position.

We must for the present bring our article to a conclusion; on some future occasion, we may again revert to the unpublished views of Dr. Wigan.

